# Students' Attitudes Toward Online Music Education During the COVID 19 Lockdown

**DOI:** 10.3389/fpsyg.2021.753785

**Published:** 2021-12-17

**Authors:** Mǎdǎlina Dana Rucsanda, Alexandra Belibou, Ana-Maria Cazan

**Affiliations:** ^1^Faculty of Music, Transilvania University of Braşov, Braşov, Romania; ^2^Faculty of Psychology and Education Sciences, Transilvania University of Braşov, Braşov, Romania

**Keywords:** music e-learning, remote teaching, perceived utility, satisfaction toward online learning and teaching, intolerance of uncertainty

## Abstract

Given the current pandemic context generated by COVID 19, important changes in the way specific subjects to music education are taught emerged, affecting not only the particularities of learning and teaching in individual courses, but also the other courses regarding group learning or theoretical subjects. In this time, emergency remote teaching and learning requires cross-collaboration between instructional, content, and technological teams. Our research examines the students' attitudes toward online education, also presenting proposals for optimization and efficiency. The research was undertaken after an experience of a University semester in a lockdown context, and it aimed at undergraduate and master's degree students from music faculties in Romania. An important result was the mediating role of perceived utility of e-learning methods, perceived utility mediated the associations between compatibility of online methods and satisfaction toward the use of e-learning methods. The perceived compatibility of e-Learning methods with online music education led to a higher perceived utility which, in turn, predicted a higher satisfaction toward e-Learning Although this period accentuated the fear of interaction with others, the anxiety related to the unknown, the intolerance of uncertainty did not predict the satisfaction toward the use of e-learning platforms. In conclusion, more educational initiatives are needed to promote remote teaching methods in music education. In the absence of similar research in our country, we considered that future research on this topic is needed.

## Introduction

The global COVID-19 pandemic brought about radical changes to all aspects of our lives and required a dramatic paradigm shift in the way we interact with each other. Social distancing and imposed restrictions influenced in-person education, especially affecting music education. Thus, as concerning music education in Romania, higher education music institutions were forced to adapt and evolve. As in many universities around the world, in Romania, too, we witnessed an unprecedented migration from the face-to-face music education to remote teaching methods, to ensure the normal functioning of education and to constrain the spread of a transmissible virus such as COVID-19.

The pandemic period brought into question the concept of Emergency Remote Teaching and Learning or Forced Remote Teaching, as students and teachers neither actively choose nor were they prepared for what was coming. Recent studies talk about students' readiness for remote learning, their experiences and self-regulation during this period (Biwer et al., 2021; Jordaan and Groenewald, 2021; Naujoks et al., 2021) and also the challenges encountered (Hoss et al., 2021; Laher et al., 2021) and students' perceptions (Gillis and Krull, 2020). Teachers were also part of the Emergency Remote Teaching process, and their experience is discussed in recent articles (Alqabbani et al., 2020), as well as compared to the students' perceptions (Zhao et al., 2021). In one article, the purposes was to explore the ways that pandemic conditions have affected music teachers' sense of safety at work and their current teaching situations, and conclusions indicate that the teachers' well-being has gotten worse from the start of the pandemic through the start of a new school year in fall 2020 (Parkes et al., 2021).

For students in higher music education, aspiring to a professional career in music, teaching and—practical, individual or group—learning activities are of utmost importance. As noted in previous research (Sleator, 2010), there are three different approaches to online education: asynchronous learning activities (Bayley and Waldron, 2019), which involve guided independent study on specific assignments, synchronous learning activities (Dammers, 2009) meaning videoconferencing on a platform in real time and blended learning, meaning the combining of instructional strategies and methods. All three approaches were encountered in the case of Romanian students.

Until the outbreak of the pandemic, in Romania, University music courses took place face to face. With the COVID 19 pandemic, the transition to remote teaching led “to a loss of classroom awareness and social presence from both the instructor and student perspectives (Nortvig and Balle, 2018). In previous studies, the advantages, and disadvantages of online education, already found in this research, have already been mentioned. Thus, among the advantages there are mentioned: the facilities offered by e-learning platforms—messages, emails, board discussion, file sharing, recording, chat, forums, better time management due to flexible hours, access from any location, and gain of time on transport (Welker and Berardino, 2005; Albert, 2014; Koutsoupidou, 2014; Schmidt-Jones, 2017; Hodges et al., 2020). On one hand, the disadvantages concern teachers, who had to renew their teaching offer rapidly and adjust their goals and activities to maintain student involvement and motivation (Wexler, 2020). On the other hand, for students this period meant the lack of in-person interaction, solving technical requirements (IT equipment, connectivity of networks) especially in the case of students in high-poverty (Warschauer et al., 2004) and rural environment (Sundeen and Sundeen, 2013). A challenging aspect could be also the need to adjust to a new study environment and to the absence of the non-verbal and immediate communication with teachers which could negatively impact the students' performance (Roseth, 2020).

Proposals will be formulated for the optimization and efficiency of online education, starting from the analysis of University students' attitudes toward online learning and at the same time, there will be presented the advantages and disadvantages of teaching and learning music online, using audio tools and/or video conferencing (e.g., Skype, MS Teams, Google Meets, Zoom, Jitsi, Big Blue Button etc.), based on the opinions and experiences of students involved in these activities.

The current pandemic context has emphasized the fear of interaction with others, the anxiety related to the unknown (Erceg et al., 2020; Harper et al., 2020) even in the case of students who were in a generally good, optimistic state (students' well-being impacting the overall learning and academic achievement). For this reason, in this study we analyzed the relationship between students' academic performance and the intolerance to uncertainty during the COVID 19 pandemic period, given that few studies that have addressed this issue. In the absence of similar research in our country and in order to outline an overview of the subject, we considered a research to address the issues of higher music education to be necessary.

The purpose of this study is to investigate the music students' attitudes toward remote education during the COVID 19 pandemic: the satisfaction toward online learning, the amount of time spent learning online, and the perceived utility and compatibility with specific subject matters. We also aimed to identify differences between the perceived utility of online learning facilities for different music education domains. Another aim was to analyse the associations between the tolerance to uncertainty and music students' attitudes toward online learning during the COVID 19 pandemic (positive and negative attitudes, perceived compatibility of e-Learning methods with online music education) and to highlight the mediating role of perceived utility of e-learning methods in the prediction of the satisfaction toward the use of e-learning methods.

## Online Music Education During Covid 19 Pandemic

A first parameter pursued in this paper aimed at the extent to which online education is appropriate for the specific of music subject matters, in the students' perception. Most previous research present online music education as an additional tool and not an exclusive one, neither inferior nor superior to traditional coursework (Groulx and Hernly, 2010). Teachers were encouraged to use and explore the effectiveness of technology resources, all in a context where they were not forced to give up traditional education. This led to a predominantly positive attitude toward technological means, reflected in the advantages and challenges of synchronous and asynchronous learning (Kenny, 2013; Dye, 2015). These means, considered modern, have a great potential to improve music education (Pike and Shoemaker, 2013; Randles, 2015; Pike, 2020), but the negative effects in the case of both exclusive and imposed long-term use have not been evaluated. Studying several music education syllabi offered by US universities, Groulx and Hernly (2010) concluded that “these online degrees are neither inferior nor superior to traditional coursework but rather represent an additional tool a University may employ to reach and educate its ever-expanding community” (p. 60).

Due to the specifics of music higher education, there are two main categories of disciplines—each with their own characteristics and problems: theoretical lessons and performance lessons/applied skills. Theoretical lessons are easier to transmit and organize in online environment, due to the predominantly informational contents (Biasutti, 2017), unlike the applied ones, in which the emphasis is on the development of individual and group musical skills. For performance lessons, the teacher cannot correct the student's posture or finger position in online lessons and cannot approach techniques such as playing pieces for piano using four hands, which are essential for demonstrating sound, rhythm, and phrasing, but are not adaptable to the virtual context. For the collective performance lessons, such as chamber music, string quartets, choirs, and orchestras, adapting to the online environment means losing the specificity of these disciplines, as this approach leads to loss of features of dynamicity, expressivity, and interactivity, all of which are crucial factors for enhancing students' performance skills.

In universities, music education teachers have no experience in distance teaching and that performing arts or composition are less adaptable to online education. According to recent research, the subject matters that are appropriate to this system would be the theoretical, group ones (music theory, composition, music history, anthropology, teacher education, aesthetics, semantics, etc.) (MacLeod, 2013; Albert, 2014; Schmidt-Jones, 2017).

In the case of children's piano lessons conducted remotely via Skype and MIDI Internet, the rewards and challenges of three different approaches to distance learning were examined: synchronous (real time), asynchronous (real time) and a combination of synchronous with asynchronous. Thus, several benefits of such lessons have been reported, but it has been stated that practical problems related to technological complications may arise (Shoemaker and van Stam, 2010; Pike and Shoemaker, 2013; Kruse, 2014; Denis, 2017).

Regarding playing a musical instrument from the full orchestra through one-to-one remote tutoring, Duffy and Healey (2017) and Crawford (2017) note that one of the limitations of this way of teaching is the lack of technical skills in both the teacher and the student, which prevent the exclusive focus on the expressive interpretation. Even in the case of students with advanced technical skills, physical interaction is necessary for their progress. Thus, an important predictor of learners' satisfaction is the perceived compatibility of the e-learning methods with the learning content in music education. Perceived compatibility is the degree to which an e-learning system is perceived as being consistent with the students' existing values, needs, and experiences and an important predictor of the behavioral intention and perceived usefulness (Sun et al., 2009).

Another parameter that needs to be observed in the online education period is the amount of time used for learning activities before the COVID 19 pandemic and during the pandemic times, in three different learning contexts (individual, group and theoretical), specific for the musical field. An important factor that influences the time parameter is experimentation before selecting the right settings for online teaching and learning, especially for adapting to musical activities, and the fact that this process is time consuming (Weiger, 2020). With reference to the relationship between time management skills and their impact on student learning and outcomes, a series of research highlight the relationship as a positive one (Krause and Coates, 2008). A 2019 study shows that effective time management is associated with greater academic performance and draws attention to the difficulties that students have in finding a balance between personal life and professional activities (Adams and Blair, 2019).

Teachers and instructors may consider extending instruction of time management skills to beyond the vocational domain, especially in the online music context, when the time allocated to individual study is combined with the time spent to meet the specific technological requirements of online education (DiPipi-Hoy et al., 2008). So far, we have not identified any research that compares the time allotted by the student for the preparation of practical or theoretical activities before and after the pandemic. A study that notices the importance of time management skills on an online learning platform mentions (exclusively online) time consuming activities: personal account management activities, accessing online courses, downloading and uploading media and learning tasks (Glava and Glava, 2012).

A third parameter pursued is intolerance to uncertainty, which has been defined as a dispositional characteristic based on perceptions, interpretations and cognitive, emotional and behavioral responses to uncertainty (Koerner and Dugas, 2006). A high level of intolerance to uncertainty can directly lead to concern, through an increased sensitivity to uncertain situations and events, which are ubiquitous.

The lack of in-person interaction between teachers and students can generate different degrees of intolerance to uncertainty, which research has shown to directly influence the achievement of academic or professional performance. In this regard, a number of studies have shown that anxiety is positively associated with performance when controlling anxiety levels as a trait (Siddique et al., 2006) especially in those individuals with a high level of skills (Perkins and Corr, 2005). Also, intolerance to uncertainty is involved in several psychological disorders, such as panic disorder, social anxiety, depression, eating disorders, obsessive compulsive disorder (Shihata et al., 2016; McEvoy et al., 2019). Another study concludes that students studying medical, technical and science faculties show a lower level of intolerance of uncertainty than students in humanistic faculties (Moffett et al., 2021).

Research shows that utility and satisfaction are major factors explaining the success of learning systems and educational effectiveness (Virvou and Katsionis, 2008; Al-Samarraie and Saeed, 2018). Perceived utility is an essential factor for increasing learners' self-regulation in e-learning environments, and increasing it also enhances learners' self-regulation toward e-learning (Tsai, 2009; Muslim et al., 2014). The perceived utility is the degree to which a user (a student in this case) of a particular system believes that it would improve their study performance as compared to alternative methods (Rizun and Strzelecki, 2020). Previous research showed that perceived utility or usefulness is positively associated with user satisfaction (Amin et al., 2014). Satisfaction toward e-learning can be defined as a sum of interactive experiences influenced by affective components generated by the human-computer interaction (Lindgaard and Dudek, 2003). Attractiveness, interactivity, and compatibility of online applications with the learning content enhance learner satisfaction (Liu et al., 2009).

The hypothesis guiding this inquiry were the following:

H1. The compatibility and perceived utility of the e-learning methods for the music education differ for different learning contexts, individual, group, and theoretical subject matters, being significantly higher for individual and theoretical than for group contexts.
H2. The amount of time dedicated to study before and during the COVID 19 pandemic varies for the three different learning contexts (individual, group, and theoretical).
H3. Does the intolerance of uncertainty negatively predict satisfaction toward the use of e-learning platforms?
H4. The perceived utility of e-learning methods mediates the associations between compatibility of remote teaching methods, intolerance of uncertainty, and satisfaction toward the use of e-learning methods.

## Methods

### Participants and Procedure

A cross-sectional design was conducted. The sample consisted of 220 students (155 female and 65 male), with a mean age of 26.57 years (SD = 8.46), from eight universities from the total of 12 universities that include music programs in Romania. We used convenience sampling, the questionnaires were implemented in the Google Forms app and sent via online methods (Facebook pages and Facebook groups for music students; University teachers from different universities were asked to distribute the call for participants to their students. With the universities from which the respondents were selected, there is a history of collaboration in research.

The questionnaires were administered online, during the second semester of the 2020 academic year and were kept active for 1 month. The questionnaires were administered without obtaining any personal, identifying information and without signing in to their Google accounts in order to ensure anonymity. Participation in the survey was fully voluntary and written consent was obtained from each participant. The objectives of the study, confidentiality of individual information, and other ethical considerations mentioned in the survey guidelines were explained to the participants prior to data collection. The participants who gave their consent that they read all the information and that they understood and agreed with all the information were able to access the questionnaires. Those who did not gave their consent, were not provided the completion link. The council of the Faculty of Music and two experts from the Faculty of Psychology and Education Sciences [name of the University was deleted for anonymity] approved the procedures for this study.

### Measures

The satisfaction of students toward the online learning and teaching was measured for three types of didactic activity organization: individual, group and theoretical subject matters. Three items were used for each of the three contexts: How do you feel about individual/collective/theoretical classes? the answers being measured on a five-point Likert scale, ranging from totally useless to extremely useful. Two additional items measured the satisfaction toward the online learning in general and toward the e-learning platforms used in the didactic activities (How satisfied are you with the online learning/ with the e-learning platform?), measured on a five-point Likert scale, ranging from not at all satisfied to totally satisfied. Another item measured the students' perception about the contribution that online learning had to the development of their competences (How much do you believe that online courses contributed to your preparation as a music student?), a five-point Likert scale also being used for the answers, ranging from a very small extent to a very large extent.

The amount of time used for learning activities before the COVID 19 pandemic and during the pandemic was also measured for the three learning contexts, individual, group, and theoretical (How much time did you spend preparing for a subject matter a week before the isolation period? vs. How much time did you spend on average preparing for a subject matter a week during this period?).

The students' perception on the compatibility of the subject matters with the online teaching and learning methods was measured for 17 subject matters classified in theoretical (e.g., music history, music theory, musical forms and analysis, etc.) and group subject matters (e.g., orchestra, choral ensemble, directing, etc.). The 17 items were measured on a five-point Likert scale, ranging from totally disagree to totally agree.

The perceived utility of online learning methods in music education was measured with a 16-item questionnaire, 8 items measuring the positive aspects of online learning methods (examples of items: The teachers are more involved in teaching, I have more time to study, etc.), Cronbach's Alpha for this dimension being 0.90 and 8 items measuring negative aspects (examples of items: I am not motivated, I have too many things to do at home), the Cronbach Alpha being 0.79. The sixteen items were measured on a five-point Likert scale.

The attitudes toward the e-learning applications used during the COVID 19 pandemic were measured with 15 items grouped into two categories, enjoyment (positive attitudes) (7 items, Cronbach's Alpha of 0.88, examples of items: it seems fascinating to use IT, I am enthusiastic about learning online) and the opposite, dislike (negative attitudes) (8 items, Cronbach's Alpha of 0.87, examples of items: I do not like it when the teacher is not next to me, I do not get feedback, etc).

The tolerance to uncertainty was measured with the Short Version of the Intolerance of Uncertainty Scale (Carleton et al., 2007). The scale measures responses to uncertainty, ambiguous situations, and the future. The 12 items measured on a five-point Likert scale ranging from 1 (not at all characteristic of me) to 5 (entirely characteristic of me) can be grouped into two dimensions, Prospective anxiety (7 items, Cronbach's Alpha of 0.89) and Inhibitory anxiety (7 items, Cronbach's Alpha of 0.87).

### Data Analysis

The measurement model through Confirmatory Factor Analysis was tested using AMOS 23.0. After performing the model modification procedures, the fit indices shod a relative good fit for the measurement model: CMIN/ df (minimum discrepancy) = 1.96, CFI (Comparative Fit Index) = 0.884, RMSEA (Root Mean Square Error of Approximation) = 0.063. Descriptive statistics were computed to check the normality of the distributions, and also to analyse frequencies for different dimensions regarding students' perceptions on online learning. In order to analyse the differences between the two learning contexts, before and after the COVID 19 pandemic for different subject matters and for aspects such as satisfaction and attitudes toward online learning and the amount of time spent for learning, *t*-tests, and repeated measure ANOVA tests were used. The normality assumption was met for all variables. Mauchly's test showed that sphericity assumption was met for all the repeated ANOVA tests. Pearson correlation coefficients were also computed to analyse the associations between variables. SPSS 23.0 was used. A mediation model was also tested, using AMOS 23.0 to determine the pathways by which perceived compatibility and tolerance of uncertainty (predictors) predicts satisfaction toward online learning (predicted variables), perceived utility being the mediator. A model with both latent variables was tested using a covariance matrix as input and maximum likelihood estimation to obtain a comprehensive view of the nature of the associations between the predictors and the predicted variable. The mediation model had excellent fit indices: CMIN/ df (minimum discrepancy) = 1.45, CFI (Comparative Fit Index) = 0.988, RMSEA (Root Mean Square Error of Approximation) = 0.046.

## Results

The descriptive analysis showed that students had medium levels of satisfaction toward the use of e-learning platforms and online education in general (27.3%); 34% of students were satisfied and 15% very satisfied, while only 33.7% reported lower levels of satisfaction. However, most of them thought that the online education during the COVID 19 pandemic had a high contribution to their professional development, as follows: 20% considered that online education had very high contribution, 24.5 a high contribution, 27.7 a medium contribution, while 27.8% considered that online education had a low (17.3%) and a very low contribution (10.5%).

To test the differences between the perceived utility of the online learning methods in different contexts (individual, group, and theoretical study) and the compatibility of e-learning methods with the specific characteristics of music education, we used repeated measures ANOVA (Table 1). The repeated measures ANOVA revealed significant differences for the two dimensions, the perceived utility being significantly higher for the individual subject matters than for group subject matters, and significantly higher for theoretical subject matters than for individual and group subject matters. The same pattern was obtained for the compatibility of e-learning methods with music education, showing that online learning and teaching methods were perceived as more appropriate for individual and theoretical study than for group study.

**Table 1:** Differences between the perceived characteristics of online learning methods in different contexts and between the amounts of time dedicated to study for the three different contexts.

	**Bonferroni comparisons**	***M (SD)***	***M (SD)***	***Mean diff*.**	***F***	***η^2^***
**Characteristics of online learning**						
Perceived utility	Individual—group	3.09 (1.38)	2.57 (1.42)	0.51^***^	61.38^***^	0.21
	Individual—theoretical	3.09 (1.38)	3.34 (1.27)	−0.25^***^		
	Group—theoretical	2.57 (1.42)	3.34 (1.27)	−0.76^***^		
Compatibility	Individual—group	2.43 (1.30)	2.19 (1.18)	0.24^***^	134.25^***^	0.33
	Individual—theoretical	2.43 (1.30)	3.18 (1.28)	−0.74^***^		
	Group—theoretical	2.19 (1.18)	3.18 (1.28)	−0.98^***^		
**Learning context**						
Individual	Before—During	3.48 (1.54)	3.52 (1.27)	−0.04	0.15	0.001
Group	Before—During	2.94 (1.49)	2.95 (1.54)	−0.005	0.002	<0.001
Theoretical	Before—During	2.56 (1.25)	2.65 (1.44)	−0.08	1.14	0.005

*F: Repeated measures ANOVA, η2: Eta squared*,

*** *p < 0.001, N = 220*.

*Adjustment for multiple comparisons: Bonferroni*.

To test if there were differences between the amount of time dedicated to study before and during the COVID 19 pandemic for the three different learning contexts, we used repeated measures ANOVA (Table 1). Mauchly's test showed that sphericity assumption was met. The results revealed that the amount of time dedicated to study does not differ between the period before the pandemic and during the pandemic, the results being similar for all the three learning contexts.

The associations between tolerance to uncertainty and perceived utility of online learning methods and attitudes toward e-learning methods highlighted significant positive associations between both prospective and inhibitory anxiety and perceived negative utility of the e-learning methods and negative attitudes toward online education, showing that higher levels of anxiety (high intolerance of uncertainty) was associated with negative attitudes and negative perceptions toward online learning methods and e-learning platforms (Table 2). As expected, a positive perception about the online music education was associated with high positive attitudes, while negative perceptions were associated with negative attitudes.

**Table 2:** Pearson correlation coefficients between tolerance of uncertainty, perceived utility, and attitudes toward online learning.

	**1**	**2**	**3**	**4**	**5**	**6**	**7**	**8**	**9**
1 Prospective anxiety	1								
2 Inhibitory anxiety	0.867^***^	1							
3 Perceived positive utility	−0.122	−0.079	1						
4 Perceived negative utility	0.366^***^	0.334^***^	−0.393^***^	1					
5 Negative attitudes	0.407^***^	0.347^***^	−0.387^***^	0.613^***^	1				
6 Positive attitudes	−0.110	−0.109	0.609^**^	−0.254^***^	−0.353^***^	1			
7 Time spent online	−0.088	−0.061	0.023	−0.008	−0.003	−0.029	1		
8 Compatibility	−0.115	−0.116	0.584^***^	−0.397^***^	−0.422^***^	0.544^***^	−0.055	1	
9 Satisfaction	−0.174^***^	−0.158^*^	0.436^***^	−0.359^***^	−0.320^***^	0.248^***^	0.133^*^	0.415^***^	1

* *p < 0.05*,

** *p < 0.01*,

*** *p < 0.001, N = 220*.

Given the associations between variables and in order to avoid multicollinearity, a mediation model was tested; the attitudes toward the e-learning applications used during the COVID 19 pandemic—positive and negative attitudes—were not included in the model because of their high associations with the perceived utility; the exogenous variables were intolerance of uncertainty (in order to avoid multicollinearity, the mediation model included the total score of the Tolerance of Uncertainty Scale, because the due dimensions of the scale were highly correlated), perceived compatibility of online methods with music education and time spent online; perceived utility of e-learning methods was the mediator, and the predicted variable was the satisfaction toward the use of e-learning methods and platforms.

**Figure 1:**
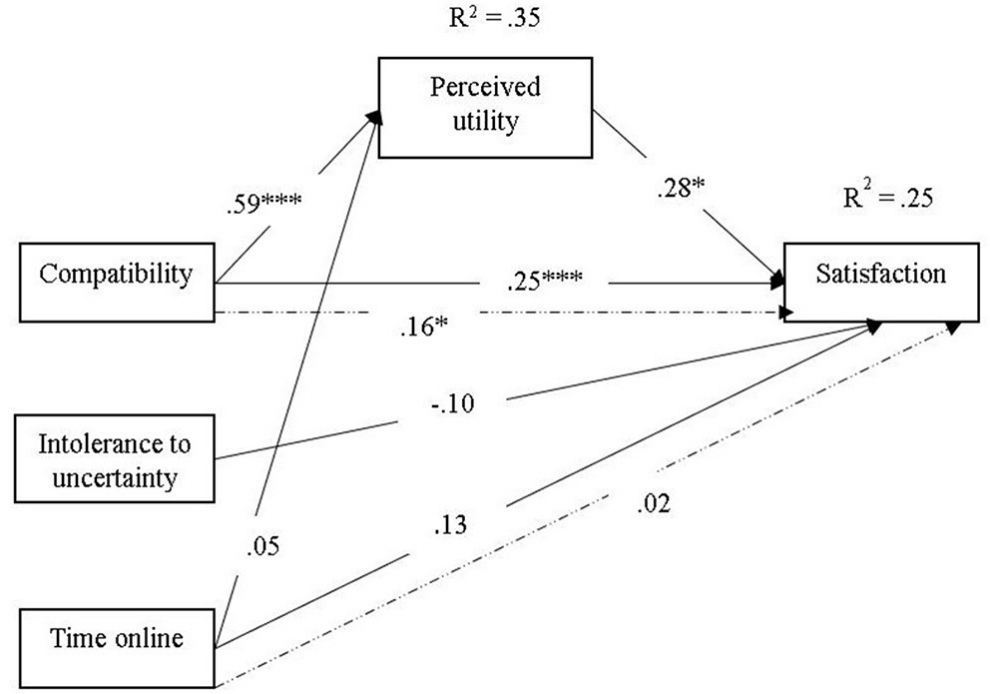
Mediation model for the prediction of satisfaction toward the use of e-learning methods and platforms (*p < 0.05 ***p < 0.001). → Direct effect ⤏ Indirect effect.

The mediation model for the prediction of satisfaction toward the use of e-learning methods and platforms revealed positive direct effects on the compatibility of online methods for the music education contexts and perceived utility, showing that a higher compatibility and a higher perceived utility would predict higher levels of satisfaction. Intolerance of uncertainty had no significant effects on satisfaction (β = −0.10, *p* = 0.070) (Figure 1). Compatibility of online methods and Time spent online predicted 35% of the total variance of the perceived utility, while Perceived utility, Compatibility of online methods, Intolerance of uncertainty and Time spent online predicted 25% of the Satisfaction toward the use of e-Learning methods. Time spent online had also significant positive effects on satisfaction (β = 0.13, *p* = 0.023) but no significant effects on perceived utility (β = 0.05, *p* = 0.318), the higher the amount of time spent learning online, the higher the satisfaction, the indirect effect of time spent online not being significant either (β = 0.02, *p* = 0.285). Perceived utility had significant positive effects on satisfaction (β = 0.28, *p* < 0.001). However, the relationship between the compatibility of online methods and satisfaction toward the use of e-learning methods was mediated by the perceived utility, the indirect effect being positive and significant (β = 0.16, *p* = 0.011) (Table 3). Perceived compatibility of online methods had positive significant effects both on Perceived utility (β = 0.59, *p* < 0.001), and on Satisfaction toward the use of e-Learning methods (β = 0.25, *p* < 0.001). Thus, a higher perceived compatibility led to a higher perceived utility which in turn, led to higher student satisfaction toward the use of e-learning facilities. The mediation was partial, given the significant direct effect of compatibility on satisfaction.

**Table 3:** Path estimates and explained variance for the satisfaction to use online methods.

	***Path***	***p***	***R^**2**^***
Predicting perceived utility			0.35
Compatibility of online methods	0.59	<0.001	
Time spent online	0.05	0.318	
Predicting satisfaction toward the use of e–Learning methods			0.25
Perceived utility	0.28	<0.001	
Compatibility of online methods	0.25	<0.001	
Intolerance of uncertainty	−0.10	0.070	
Time spent online	0.13	0.023	
Indirect effect of compatibility through Perceived utility	0.16	0.011	
Indirect effect time spent online through Perceived utility	0.02	0.285	

*N = 220*.

## Discussion And Conclusions

In the Romanian music faculties, the curriculum included several types of subject matters: theoretical subject matters, studied in large groups and practical subject matters, which can be studied individually (teacher-student), in small groups (chamber music with 2–5 instrumentalists) or in large groups (orchestra, choral ensemble). Although the specifics of the theoretical disciplines in music lie in both theoretical lectures and interactive practical activities, the transition to the online environment did not greatly affect their development, because the online platforms used contained specific teaching aids.

In the case of individual and group practical subject matters, problems have arisen such as: video and audio quality because of the platforms or the quality of personal devices, lack of physical proximity and eye contact (Dammers, 2009; Orman and Whitaker, 2010), internet connection, environmental conditions conducive to study, lack of personal musical instruments, impossibility of synchronous performance because of technical conditions, which led to a forced adaptation/replacement of specific activities (e.g., chamber music, orchestra, choir, opera classes). As public concerts and auditions were no longer possible during the pandemic, they were replaced by individual audio and video recordings made by students in their own homes. The individual recordings were also made for the disciplines that are studied in small or large groups, later they were overlapped and mastered in one performance. The lack of in-person interactions between musician-audience or learner-teacher and the absence of students' performative experience, were major aspects that could not be transferred to the virtual environment.

Our study confirmed the fact that for the theoretical and individual subject matters the online teaching and learning methods could be more efficient and more appropriate for music education than for individual subject matters, the first hypothesis being supported by the data obtained. Therefore, teachers could consider using e-learning platforms for theoretical subject matters in music education as an opportunity for increased musical exchange and cultural interaction (Juklová et al., 2017; Cho, 2021). The low perceived efficiency for group subjects could be explained by the fact that the online context limits the interpersonal experiences leading to a sense of loss of social connections due to the lack of socializing opportunities and joint music-making (Levstek et al., 2021). Although previous research has demonstrated the effectiveness of online teaching in the case of instrument lessons, or in terms of creative, group music activities (Pike and Shoemaker, 2013; Kruse, 2014; Denis, 2017), our study demonstrates that online music education is a necessary and useful tool, but it can also bring a series of risks. We believe that for individual instrument lessons or for practical courses in orchestra, choral ensemble or chamber music, online education can only be an additional, occasional form that can complement, not replace, the in-person forms of education, where students interact with each-other and with their teachers. The lack of the possibility to perform rehearsals for choir, orchestra, band, chamber music during the pandemic was a phenomenon felt internationally. Because of this, alternative methods of supporting group music activities have been developed, replacing them with individual practices, which were then brought together through technology. These practices of individual recording, subsequent overlapping of recordings and their simultaneous rendering have brought benefits such as: the opportunity to participate from everywhere, the students' possibility to show their personalities and to work more on their individual parts, to perform without stage fright, but we cannot deny the obvious disadvantages in terms of lack of collective musical activity. The students were forced to renew their collaborative effort, learning to work together in a new environment.

Concerning the second hypothesis, the amount of time dedicated to study before and during the COVID 19 pandemic does not differ for the three different learning contexts (individual, group, and theoretical), thus the hypothesis was not sustained. Regarding this hypothesis, because many University students work full-time or part-time, access to online courses and programs gives these students greater flexibility in planning their course completion strategy. Online education is more accessible than on-campus courses, given the costs and travel time. On the other hand, we aimed to test this hypothesis to see whether the individual activity carried out on the e-learning platform, with all its technical requirements, extended the time assigned to study, compared to the period before the pandemic, and also if there are significant differences between the three learning contexts, as in the case of the perceived utility of the online learning methods. Checking the parameter related to the time allotted to the study is important, especially from the perspective of the relationship between the study time and performance level (Jørgensen, 2002). So, the time that the student assigns to individual study affects the progress that the student will have. As other studies also showed, in general, students experienced more difficulties in managing their time and regulating their learning than before the shift to online education, students investing more time and effort in their self-study (Biwer et al., 2021). The technology for supporting online music education is not only about using the e-learning platform, but also plug-ins/softwares for audio/video recording, and becoming confident in using them is very time consuming (Biasutti et al., 2021).

However, despite the context of uncertainty generated by the COVID 19 pandemic the third hypothesis was not supported by the data, the results showing that the intolerance of uncertainty does not predict the satisfaction toward the use of e-learning platforms. An important result of our study was the mediating role of perceived utility of e-learning methods, perceived utility mediated the associations between compatibility of online methods and satisfaction toward the use of e-learning methods, the fourth hypothesis being sustained. In line with previous studies, our results confirmed that the perceived compatibility of e-learning methods with the learning content in music education was a significant positive predictor of perceived usefulness of e-learning platforms and of learners' satisfaction (Venkatesh et al., 2004; Sun et al., 2009). Concerning the perceived utility of e-learning, our results confirmed previous studies in the field, showing that perceived utility is a positive predictor of learner's satisfaction (Liu et al., 2009; Liaw and Huang, 2013; Islam, 2016). Thus, if the e-learning platform is compatible with the study, this positive perception would lead to a higher perceived utility which, in turn, predicts a higher satisfaction toward e-Learning.

## Implications

The research results highlighted several advantages of online education. First, we note the accessibility and flexibility of courses, portability, and receptivity, with students having the opportunity to access courses anytime and anywhere and reduce travel, accommodation and study costs on campus. Due to the use of technology, new skills can be developed. Another aspect concerns the instrumentalists, who have to make records of their performances in order to be evaluated. Thus, through repeatability, it contributes to a better learning of the repertoire but also to a self-analysis and a more precise evaluation. Other researchers have talked about limitations, identifying a number of issues that hinder the effective teaching and learning of music (Dammers, 2009; Brändström et al., 2012; Kruse et al., 2013; Kruse, 2014). Thus, the problem of solving the technical requirements of online teaching has often been invoked, because there are situations in which the servers cannot support many users at the same time and can stop, because of overload; problems can also occur because of the lack of computers and IT equipment for students, including wi-fi in their homes.

The online is ultra-individualistic, the camera and the current communication technique emphasize only the one who speaks or responds while taking the others out of the game. As an effect of this aspect, since most of the time students are forced to turn off their microphones while the teacher speaks, boredom and lack of concentration may appear; they feel excluded from the process.

Another limitation of this learning process in the case of music education is the lack of sound quality and fidelity of sound reproduction, because of the internet connection, microphones that pick up sound and personal sound reproduction systems. Especially in the classes of chamber music, conducting or choral ensemble, there is a lack of synchronization because of the platform, which eliminates all the benefits of group performance. However, as other researchers also highlighted the COVID 19 pandemic created opportunities for teachers and students to use technology, to be more focused on individual musicianship, to focused in their teaching on music theory, history, and culture (Hash, 2020).

In face-to-face music education, teachers' body language, facial expressions, voice, and attitude are important teaching tools (Riley, 2009). A major disadvantage of sudden shift in pedagogical offers caused by the COVID-19 health emergency is the lack of in-person interaction, in all its variants, simultaneous, multiple, emotional, empirical, because education means not only pure information, but also the involvement of all senses—smell, touch, sight, hearing (Hebert, 2008). Also because of the lack of in-person interaction, there is a desocialization and lack of feedback from the students.

## Limitations And Future Research Directions

Our study has several limitations that need to be acknowledged and which could be addressed in future research. Firstly, the research was cross-sectional, causal interpretation of results not being possible. It would be useful to test the validity of the proposed relationships in a longitudinal setting and in a typical online setting, and not in exceptional settings such as the emergency remote teaching generated by the COVID 19 pandemic. Secondly, there are also other important variables which could explain e-learning system use outcomes, not included in this study, such as personal characteristics (online self-efficacy, technology anxiety, user experience regarding e-learning platforms, learning engagement) (Maican et al., 2019; Stephan et al., 2019) or dimensions of the acceptance technology models (perceived ease-of-use, social influence, performance expectancy, effort expectancy or facilitating conditions) (Venkatesh et al., 2003). Future research may provide a closer look on understanding the effects of these variables on learners' satisfaction or intention to further use online applications, but also on more complex learning outcomes, such as academic performance (Nicolau et al., 2020). Future research cold also focusses on the impact of online teaching and learning and the development of self-regulated learning skills and autonomous learning of music students (Bonneville-Roussy et al., 2020) and on interventions to reduce burnout and anxiety (Grigorescu et al., 2020). Future research could include qualitative research, as the present one is also limited by the lack of it. A qualitative approach could offer insights about student engagement with online learning components, the need for peer-collaboration in online settings and teacher-student adaptability or the development of relatedness between teacher and student (de Bruin, 2021).

More educational initiatives are needed to promote online distance learning in music. As we have not yet encountered any research that refers to the practical group activities—choir, orchestra, chamber music, band—in higher education, during this pandemic, we will consider this aspect in further research.

## Data Availability Statement

The raw data supporting the conclusions of this article will be made available by the authors, without undue reservation.

## Ethics Statement

The studies involving human participants were reviewed and approved by the council of the Faculty of Music, Transilvania University of Brasov. The patients/participants provided their written informed consent to participate in this study.

## Author Contributions

All authors listed have made a substantial, direct, and intellectual contribution to the work and approved it for publication.

## Conflict of Interest

The authors declare that the research was conducted in the absence of any commercial or financial relationships that could be construed as a potential conflict of interest.

## Publisher's Note

All claims expressed in this article are solely those of the authors and do not necessarily represent those of their affiliated organizations, or those of the publisher, the editors and the reviewers. Any product that may be evaluated in this article, or claim that may be made by its manufacturer, is not guaranteed or endorsed by the publisher.
